# 3D Models Currently Proposed to Investigate Human Skin Aging and Explore Preventive and Reparative Approaches: A Descriptive Review

**DOI:** 10.3390/biom14091066

**Published:** 2024-08-26

**Authors:** Francesca Lombardi, Francesca Rosaria Augello, Alessia Ciafarone, Valeria Ciummo, Serena Altamura, Benedetta Cinque, Paola Palumbo

**Affiliations:** 1 Department of Life, Health and Environmental Sciences, University of L’Aquila, 67100 L’Aquila, Italy; francesca.lombardi@univaq.it (F.L.); francescarosaria.augello@univaq.it (F.R.A.); alessia.ciafarone@graduate.univaq.it (A.C.); serena.altamura@graduate.univaq.it (S.A.); benedetta.cinque@univaq.it (B.C.); 2 Department of Innovative Technologies in Medicine and Dentistry, University “G. d’Annunzio” of Chieti-Pescara, 66100 Chieti, Italy; valeria.ciummo@phd.unich.it

**Keywords:** human skin, 3D skin models, aging, pseudo-3D system, skin organoids, skin-on-chip, reconstructed human skin, 3D bioprinting, skin microbiota

## Abstract

Skin aging is influenced by intrinsic and extrinsic factors that progressively impair skin functionality over time. Investigating the skin aging process requires thorough research using innovative technologies. This review explores the use of in vitro human 3D culture models, serving as valuable alternatives to animal ones, in skin aging research. The aim is to highlight the benefits and necessity of improving the methodology in analyzing the molecular mechanisms underlying human skin aging. Traditional 2D models, including monolayers of keratinocytes, fibroblasts, or melanocytes, even if providing cost-effective and straightforward methods to study critical processes such as extracellular matrix degradation, pigmentation, and the effects of secretome on skin cells, fail to replicate the complex tissue architecture with its intricated interactions. Advanced 3D models (organoid cultures, “skin-on-chip” technologies, reconstructed human skin, and 3D bioprinting) considerably enhance the physiological relevance, enabling a more accurate representation of skin aging and its peculiar features. By reporting the advantages and limitations of 3D models, this review highlights the importance of using advanced in vitro systems to develop practical anti-aging preventive and reparative approaches and improve human translational research in this field. Further exploration of these technologies will provide new opportunities for previously unexplored knowledge on skin aging.

## 1. Introduction

To effectively study skin aging, it is essential to have suitable models. Over the years, in vivo, in vitro, and in silico approaches have been developed for pharmaceutical and cosmetic testing. Due to growing ethical and economic concerns, alternative in vitro models have been designed to replace or reduce animal experiments guided by the ethics of the ”3 R” principles: replacement, reduction, and refinement [1]. These “principles” were formulated to assist researchers in discovering and using the latest available techniques and to encourage the development of new tools and methods, defining the fundamental objectives of an innovative science [2]. Animal models are subject to ethical restrictions and show noticeable genetic differences from humans. These issues have further increased the need for alternative in vitro systems mimicking natural skin’s structural and functional hallmarks. Therefore, the development of alternative models has gained a high priority following the European ban on animal testing for cosmetic products (2009/1223/EU), the REACH guideline for chemicals (2006/1907/EC), and the recommendation to follow the 3 R principle for research (2010/63/EU) [3].

Overall, the technical advances of 3D models may aid in uncovering the underlying causes of skin aging, which may further fuel the discovery of suitable treatments. The aim of this review is to describe the 3D experimental models currently proposed for investigating the molecular mechanisms underlying human skin aging [4] (i.e., upregulation of stress regulatory proteins, reactive oxygen species (ROS) accumulation, defects in integrin expression, increased collagen breakdown, telomere shortening, secretory-associated senescence phenotype (SASP)) and exploring preventive and reparative approaches.

## 2. From Basic 2D to Complex 3D Models

Two-dimensional in vitro models are a cornerstone in biomedical research, since they allow recreating a simple and controllable environment to study cellular behavior, drug responses, and disease molecular mechanisms. They also show the important advantages of being cost-effective and easy to maintain. By providing an accessible and economical platform and representing a quick and repeatable model to evaluate skin responses, 2D models facilitate experimental replication and high-throughput screening, making them indispensable in the initial research and drug development phases [5,6,7].

However, the currently used in vitro 2D skin aging models have no negligible limitations. Firstly, they do not fully replicate the complex structure and functionality of human skin because they lack its three-dimensional structure, which includes distinct layers such as the epidermis, dermis, and subcutaneous tissue. Particularly, the aged skin undergoes various structural and biochemical alterations that 2D models cannot absolutely replicate. These features, such as the spatial 3D organization, can affect the accuracy of experimental outcomes. Moreover, 2D models fail to mimic the dynamic interactions between skin cells, immune cells, and the extracellular matrix [8].

One of the crucial functions of the skin is to act as a barrier against environmental factors, another item improperly replicated by 2D models, limiting studies on permeability and drug delivery. The 2D cells interact only with nearby cells within the horizontal plane and are uniformly and directly exposed to stimuli or drugs without the action of other cellular types, limiting their ability to reproduce chemical and biological factors, i.e., the pH and oxygen gradients of the in vivo microenvironment, and cellular interactions [9]. The limitations in mimicking the true barrier function and tissue environment of human skin can result in unreliable data regarding drug absorption, efficacy, and toxicity when using 2D models. Also, mechanical properties of the skin, such as firmness and elasticity, are not replicated in 2D models. Aging skin physiologically changes in these properties, and the inability to mimic them accurately in 2D models can affect the relevance of experimental outcomes. In the dermis, human skin aging is characterized by reduced collagen synthesis, decreased mitochondrial function, lower ATP levels, and increased nuclear factor-kappa B (NF-κB) signaling, which is associated with inflammation and damaged skin barrier function [10]. The absence of immune cells and blood vessels in 2D models strongly limits the study of inflammatory and vascular responses in the skin aging process [11].

**Figure 1:**
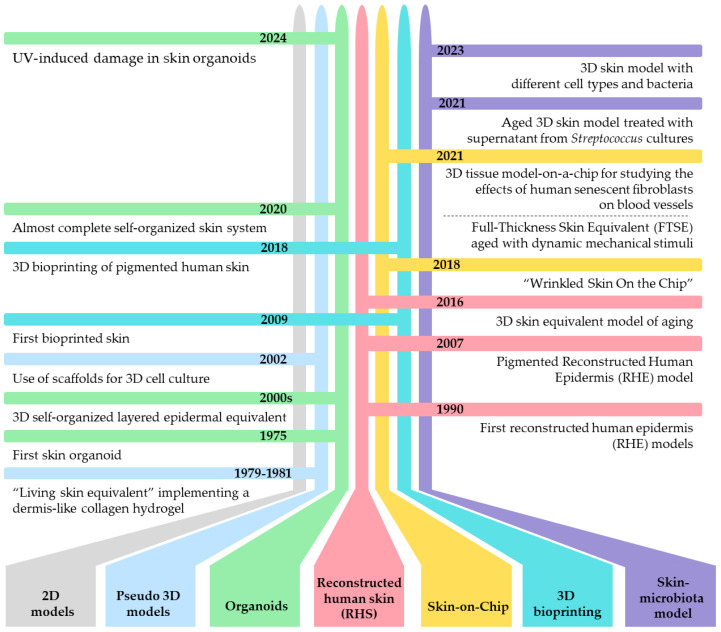
Timeline of the different advances in the development of skin 3D models. Pseudo-3D models: Bell et al., 1979 [14], 1981 [15,16]; Li et al., 2002 [17]. Organoids: Rheinwald et al., 1975 [18]; Itoh et al., 2011 [19], 2013 [20], Guenou et al., 2009 [21], Takahashi et al., 2006 [22]; Lee et al., 2020 [23]. Reconstructed human skin (RHS): Fentem and Botham 2002 [24]; Liu et al., 2007 [25]; Diekmann et al., 2016 [26]. Skin-on-chip: Lim et al., 2018 [27]; Jeong et al., 2021 [28]; Pauty et al., 2021 [29]. Three-dimensional bioprinting: Lee et al., 2009 [30]; Min et al., 2018 [31]. Skin microbiota model: Kim et al., 2021 [32]; Rikken et al., 2023 [33].

Moreover, due to their simplified structure, 2D skin models may not be suitable for long-term studies, as they often fail to maintain cellular viability and function over extended periods, which is essential for chronic toxicity and prolonged treatment studies. Additionally, cells in 2D cultures can often exhibit different morphology, proliferation rates, and differentiation patterns compared to those in vivo. Hence, although the 2D skin models are generally less expensive and easier to be established in cell biology research and easy to be analyzed by several molecular, biochemical, and image-based assays than 3D models, they may not always provide the necessary accuracy for studies of translational relevance. This can lead to higher costs and efforts in later stages of research and development. All these limitations highlight the need for more advanced and realistic skin models, such as 3D organotypic cultures and “Skin-on-Chip” technologies [12,13], that could better replicate human aged skin for research purposes to transfer the results to the clinic. The timeline in Figure 1 depicts the most significant advances in developing skin 3D models.

## 3. 3D Models as Innovative Tool for Studying Skin Aging

Generally, 3D cell culture models represent a significant innovation with respect to traditional 2D models and serve as a suitable alternative to animal models in industrial applications and experimental research, providing a more accurate representation of the in vivo environment. Three-dimensional skin aged models can simulate the multi-layered structure of human skin and allow the physiological cell–cell and cell–matrix interaction in three dimensions, mimicking the natural architecture and organization of tissues. For the first time, in the middle of the 20th century, Medawar defined the method of growing adult mammalian skin epithelial cells from skin explants. He achieved this by incubating the skin epithelium in a specific medium supplemented with serum, maintained in gaseous equilibrium with air or an air-oxygen mixture, and the cells that formed it were able to proliferate and migrate [34].

Another important factor is that 3D skin models can have the simultaneous presence of both aged cells and non-aged cells as well as immune cells, vascular components, and, not least, appendages (e.g., sweat glands, sebaceous glands, hair follicles, and vessel-like channels)—all reviewed in Mori et al. [35]. These features, increasing the model complexity, enhance the soundness of experimental results and understanding of how cell communication may be involved in the aging process of tissues [36].

Developing 3D in vitro models for skin aging, recreating an aged phenotype, poses a significant issue since aging is a multifaceted process that involves numerous intrinsic and extrinsic factors and complex biological and molecular changes that are difficult to replicate in standard, shorter-term in vitro models. Overall, 3D skin aged models represent a significant advancement in studying skin aging, offering a more accurate and comprehensive representation of these complex processes [37]. Also, innovative skin aging systems provide information on approaches that could slow down the aging process by extending the healthy life span.

Since, as said before, aging is an irreversible biological process that takes decades in an organism, a crucial factor to consider for generating an aged skin model is the culture time of the cells involved. In this regard, Dos Santos et al. studied the chronological aging of the epidermis, developing an in vitro 3D model of skin aging showing the age-related phenotype (decrease in the epidermis thickness, a reduced expression of hyaluronan and its surface receptor CD44, etc.) [38]. Although this model is very similar to in vivo aged skin, the long culture time to establish it is a disadvantage for the high-throughput generation of skin equivalents [37]. To overcome this hurdle, bioengineered skin models have been created with the direct incorporation of senescent cells that stop dividing but remain metabolically active, added with other normal cells, providing a more accurate representation of the skin microenvironment and the complex interactions between all cells. It is known that senescent cells strongly influence the surrounding tissue by secreting pro-inflammatory factors, chemokines, cytokines, and proteases, known to induce the SASP [39]. The aged in vitro model (cultured skin equivalents) was generated through the co-culture of keratinocytes and senescent fibroblasts on a scaffold made of collagen, glycosaminoglycan, and chitosan. To simulate the senescence phenotype, fibroblasts were previously exposed to mitomycin-C that induced premature senescence by triggering DNA damage and producing reactive oxygen species (ROS). The mitomycin-C-treated fibroblast showed an aged-related phenotype characterized by a reduced proliferative ability, decreased expression of filaggrin, an essential protein for the correct formation and function of the skin protective barrier in the epidermis and lowered elastin and collagen in dermal tissue, and increased secretion of pro-inflammatory cytokines [26].

Moreover, to increase the efficiency of 3D aged skin models, more recent work has been carried out incorporating increasing ratios of hydrogen peroxide (H_2_O_2_)-induced premature senescent fibroblasts together with normal fibroblasts into the collagen matrix. This skin equivalent, named “*senoskin*”, recapitulated several key hallmarks of skin aging, such as reduced collagen production, increased levels of pro-inflammatory cytokines, and an altered extracellular matrix (ECM) composition [40].

Victorelli et al. suggested further improvement of 3D skin models since they incorporated ultraviolet (UV)-induced senescent melanocytes in skin equivalents. This model even more mimicked the structure and function of native human skin and allowed a better understanding of how these cells affect the microenvironment in a setting that closely resembles in vivo conditions. Senescent melanocytes expressed various inflammatory and SASP markers, which are known to induce skin aging by influencing nearby non-senescent cells and the ECM. This process induced telomere dysfunction and decreased proliferation of surrounding cells (keratinocytes and fibroblasts) through CXC chemokine ligand 3 (CXCL3)-dependent mitochondrial ROS, thereby contributing to skin aging [41].

An alternative way to develop 3D aged skin equivalent is by the incorporation of advanced glycation end-products (AGEs) into dermal–epidermal compartments. The study of Markiewicz et al. utilized a commercially available full-thickness skin equivalent (a dermal–epidermal skin model with a fully developed basement membrane (EpiDerm-FT™)) treated with methylglyoxal (MGO), a highly reactive dicarbonyl compound and a key intermediate in AGE formation, in culture media. The MGO-induced AGEs in the 3D skin model resulted in significant changes, including a rapid aged phenotype induction characterized by the increase in AGE formation, which interfered with the skin’s structural integrity, decreased collagen expression, and reduced stratification of the epidermis. Of note, in this 3D skin model, external treatment with derivatives of resveratrol reduced the AGEs’ generation, decreased the expression of aging and oxidative stress markers, and restored collagen levels and epidermal integrity [42]. Lee and colleagues established an in vitro 3D skin model from EFT-400 full-thickness reconstituted skin tissue (EpiDermFT) in which human keratinocytes and fibroblasts were sequentially cultured on a matrix used to support cell growth in three dimensions, allowing them to create the epidermal and the dermal layer, respectively. To induce the aging process through glycation, the new 3D skin was exposed to glyoxal (GO). In order to assess the degree of glycation within the skin equivalent, the levels of Nε-(carboxymethyl)lysine, a well-known marker of glycation, were evaluated. This 3D skin model, imitating the effects of glycation on skin aging, could be a good tool to screen the antiglycation activity of topical formulations, counteracting the effects of glycation and slowing the aging process [43].

Exogenous stressors, such as UV radiation, are significant environmental factors contributing to skin aging, a process known as photoaging. Only the ultraviolet type A (UVA) (320–400 nm) and ultraviolet type B (UVB) (280–320 nm) can pass through the atmosphere, reaching the skin. Direct exposure of skin to sunlight or UV radiation led to harmful effects, including sunburn, inflammation, photo-immunosuppression, and even skin cancers [44]. Additionally, UV radiation is a primary cause of premature skin aging since the UVA exposure accumulates ROS that activate the NF-κB and mitogen-activated protein kinase (MAPK) signaling pathways [45] and increase the downstream molecule tumor necrosis factor alpha (TNF-α) and matrix metalloproteinases (MMPs) expression, leading to ECM degradation (alteration in collagen and elastic fibers in the dermis). All these events can be measured in deep wrinkle formation, skin laxity, and hyperpigmentation, all features of photoaged skin [46]. In view of this, Casale et al. established a human full-thickness skin model (named Endo-HSE) exposed to UVA radiation as an advanced in vitro model to examine the downstream effects of UVA damage and test the efficacy of photoprotectants, such as sunscreens, antioxidants, and retinoic acid. In detail, fibroblasts were obtained from human tissue biopsies, cultured on gelatin microcarriers, then transferred to a maturation chamber for six weeks to form disk-shaped tissue constructs. Subsequently, to create epithelium, keratinocytes and melanocytes were plated onto a newly formed dermal layer for one week in submerged conditions and then lifted to the air–liquid interface for up to 14 days. The new 3D skin model was treated with UVA radiation by using a UVA lamp in order to simulate the solar exposure and has measured significant oxidative stress in both the dermis and epidermis and increased senescence markers expression (such as p63, Ki67, and activated caspase 3) in the epidermal layer, a reduced self-renewal capacity of skin stem cells were measured, and also the modified remodeling of collagen architecture in the dermis was notably observed, all changes associated with aging [47].

## 4. Overview of 3D Aged Skin Models

**Figure 2:**
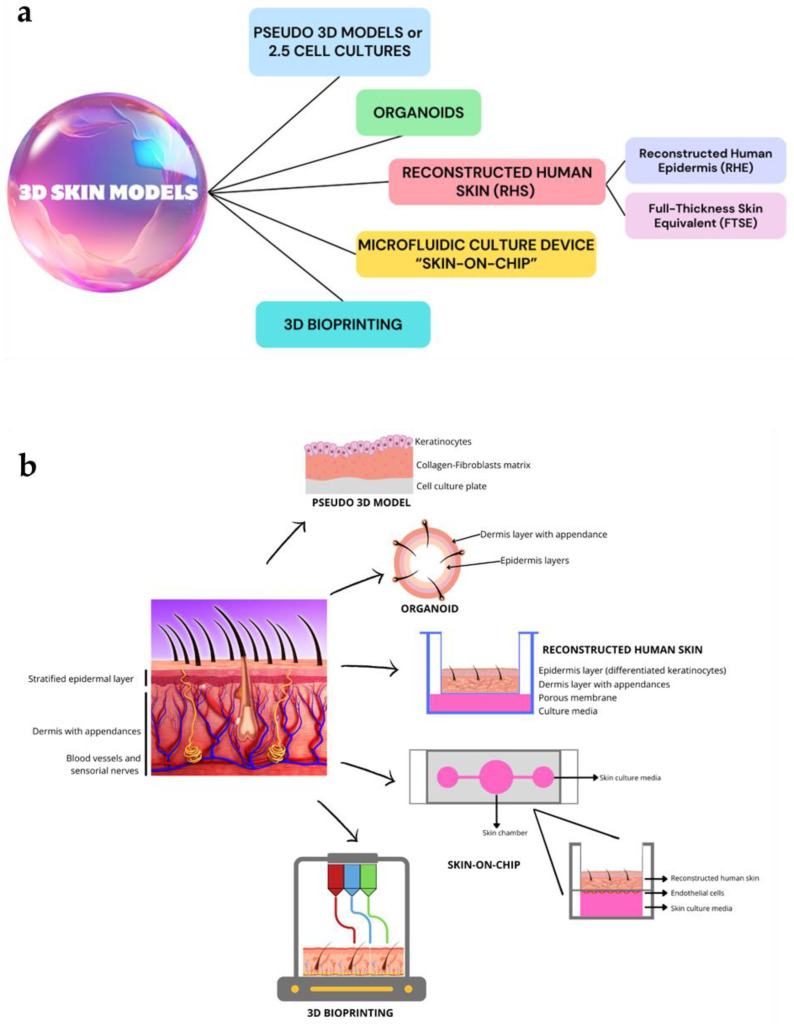
A schematic overview of the main 3D skin models (**a**) and different microstructure of skin obtained by each modeling technology (**b**). These images were created with www.canva.com, accessed on 12 August 2024.

The 3D innovative skin models, described in the following paragraphs highlighting their peculiar features, are listed in Figure 2a. Figure 2b summarizes the microstructure of obtained skin by applying these models.

### 4.1. Pseudo-3D Systems

It is known that skin aging leads to decreased elasticity, loss of dermal matrix integrity and cell contraction, and an impaired migration ability. A valid cellular model of aged skin should also consider these features, which are significantly compromised in senescent cells compared to younger ones. In this scenario, it is necessary to specify the presence of models named “pseudo-3D systems” or alternatively called “2.5D cell cultures”. These are hybrid models borderline between 2D and 3D in which cells are grown in a bidimensional condition and are plated on top of a thick layer on ECM-coated culture and, therefore, do not fully reflect the complexity of 3D systems, but at the same time, try to recreate multiple physiological conditions. However, these cultures do not entirely replicate an in vivo environment, as the cells are still embedded into the culture medium and lack direct and complete contact with the ECM on their surface. Cell migration in the 2D space is primarily driven by adhesion and traction forces. However, in a three-dimensional microenvironment, additional factors such as biochemical, steric, and mechanical influences play a significant role in in vivo stiffness and then in cell migration [48]. The reduced functional ability in terms of the cell migration of senescent fibroblasts has been recently verified using a 2.5D model. Human dermal fibroblasts (FF-95) derived from foreskin tissue were induced to express an aged phenotype following replicative senescence, and then they were mixed with a solution of type I bovine collagen to evaluate their migration. Senescent fibroblasts, when placed in 2.5D culture, exhibited a significant reduction in contractility within collagen gels compared to younger cells, highlighting their weakened mechanical properties. The contractility reduction is due to alterations in cytoskeletal organization, which are crucial for maintaining structure and function in young skin [49]. To sum up, although pseudo-3D models offer valuable insights into the behavior of aged cells, they have the critical limitation of not perfectly replicating the complexity of in vivo environments. These models have underscored the impaired mechanical properties and migration abilities of senescent fibroblasts, emphasizing cytoskeletal changes as a crucial factor in aging.

### 4.2. Organoid Cultures

Three-dimensional structures that self-organize due to the natural tendency of adherent cells to aggregate themselves, known as organoids, appear to be a significant breakthrough for scientific research. Sato et al., in 2009, created for the first time organoids from intestinal stem cells [50]. Subsequently, they have been developed from various tissues, including the lungs, brain, stomach, liver, pancreas, prostate, mammary gland, fallopian tubes, taste buds, lungs, salivary glands, esophagus, epididymis, tongue, lacrimal gland, and thyroid (all reviewed in Suarez-Martinez et al. [9]).

Organoids derive from stem cells such as induced pluripotent stem cells (iPSCs), adult stem cells (ASCs), or embryonic stem cells (ESCs), and they develop into organotypic structures when cultured in a 3D matrix with specific growth factors. This process involves stepwise differentiation protocols that mimic gastrulation and organogenesis signals, and by using various growth factors or inhibitor molecules, the formation of specific germ layers, such as endoderm, mesoderm, and ectoderm, could be induced [51]. iPSCs used to create organoids are generated by reprogramming adult cells to a pluripotent state, allowing them to differentiate into any cell type [52]. Regarding ASCs, the cells are isolated directly from skin tissue and expanded in culture and can be induced to form skin organoids by providing an appropriate 3D scaffold and a cocktail of growth factors promoting skin differentiation [53].

Notably, organoids can form lumens, gland-like structures, and other organ-specific features [23]. Hence, organoids are miniaturized organs with a 3D structure and multiple cell layers composed of tissue-specific cell types and stem cells, exhibiting a unique intrinsic organization [54]. They can be expanded over long periods, genetically modified, and cryopreserved while maintaining their phenotypic characteristics [55,56].

Organoids substantially differ from spheroids, another three-dimensional cell culture with a spherical morphology, generally composed of a single somatic cell type (including cancer cells, primary cells, or cell lines). When the attachment to a substrate is precluded, the cells are able to self-aggregate and generate the spheroid that better mimics cell–cell and cell–matrix interactions than 2D cultures, although they lack the capacity to replicate the more complex structure or function of an organ [57]. Spheroids are commonly used in cancer research to assess tumor growth, invasion, and drug resistance or are used in drug screening and in studies of cell–cell and cell–matrix interactions [58]. Of note, organoids are used in developmental biology to study organogenesis and tissue differentiation, in disease modeling to understand the mechanisms involved in different diseases, such as genetic disorders, infectious diseases, and cancer within a specific organ context, and are employed in regenerative and personalized medicine to develop and test new therapies. Finally, organoids serve as platforms for studying complex organ functions and interactions with their microenvironment [59]. Organoids derived from patient samples, known as patient-derived organoids (PDOs), have shown promise in cancer research for personalized medicine, as they can more accurately replicate the genetic landscape and drug response of the original tumors compared to traditional 2D cultures [60]. Furthermore, PDOs represent a potential preclinical model tool to identify novel biomarkers of aging and design personalized approaches for the prevention and treatment of age-related diseases [61].

Regarding the skin models, the concept of utilizing a more complex in vitro skin culture system was first introduced in 1975 by Rheinwald et al., who tested a self-organizing approach to produce stratified squamous epithelium through the serial co-cultivation of primary human keratinocytes and irradiated mouse fibroblasts. This innovative discovery paved the way for the in vitro culture of self-organizing skin tissue [18]. In the early 2000s, 3D self-organized layered epidermal equivalents obtained from ESCs and iPSCs were developed, representing a significant breakthrough in the field of skin organoids [19,20,21,22]. Now, 3D skin equivalents can faithfully replicate the right structure and composition of native skin, including the epidermis, dermis, and also skin appendages [62,63]. In 2011, Itoh et al. produced 3D skin equivalents by seeding keratinocytes obtained by fibroblast-derived iPSC and stimulated with a specific growth factor onto a support matrix containing human feeder fibroblasts [19]. The epithelial differentiation of iPSCs into keratinocytes was achieved using retinoic acid and bone morphogenetic protein 4 (BMP4) to prevent neural differentiation [64]. The organoids obtained with this experimental protocol closely resembled the complex layering of native epidermis due to the presence of stratification of iPSC-derived keratinocytes [65]. In 2020, Lee et al. generated an almost complete self-organized skin system in vitro differentiated from iPSCs, forming a skin organoid that recapitulated many appendage structures, including hair follicles [23]. Interesting studies provided valuable results into the development and functionality of skin-related organoids, specifically focusing on mature skin systems of appendages (sebaceous and sweat glands, respectively) [66,67]. The first study highlights that the expression of B lymphocyte-induced nuclear maturation protein 1 (Blimp1) on hair follicle stem cells and cells that reside in the sebaceous gland base is responsible for generating sebaceous gland organoids. These organoids exhibit key characteristics of sebaceous glands, such as lipid secretion, making them a helpful tool for researching gland biology and skin conditions like acne [66]. The second one developed functional sweat gland organoids, which are crucial for thermoregulation and skin hydration. They generated organoids from mouse sweat gland-derived stem cells cultured in a 3D matrix and specific growth factors [67].

To obtain organoids for aging research, Pitrez et al. recently described how reprogramming somatic cells into iPSCs then differentiated in specific cell types was efficient. Moreover, an alternative strategy to investigate aging mechanisms is the transdifferentiation from one somatic cell type to another obtained from aged or age-related disorder-affected donors. However, the challenges in accurately mimicking the aging process and translating findings to in vivo are still ongoing [36]. iPSC-derived skin organoids initially resemble early fetal skin structures and then develop more mature features after extended culture. However, they are not effective in simulating the complex and dynamic changes that occur during aging in vitro [68]. Therefore, based on the previous work [38], Sun et al. hypothesized that prolonging the time of the organoid culture in vitro as much as possible or obtaining skin organoids generated from fibroblasts exposed to mitomycin C could represent two more feasible methods to induce aging [69]. More recently, Kim and colleagues described in detail the procedure for obtaining skin organoids. They developed a peculiar model of skin organoids with hair follicles to evaluate the skin’s response to environmental factors like solar ultraviolet exposure [70]. Skin organoids are generated from human iPSCs, which can differentiate into various skin cell types. The process begins with the induction of iPSCs to form embryoid bodies, which are then induced to differentiate into skin cells, including keratinocytes, melanocytes, and dermal fibroblasts, using specific supplements added in the culture medium. After 85 days, the newly formed skin organoids were divided into eight equal parts, which were placed on polymerized collagen I-coated transwell culture inserts in a humidified incubator for 3–4 weeks. To promote further epidermal maturation, the skin organoids were cultured in an air–liquid interface culture condition at 37 °C for six days, replacing the medium daily. To induce photodamages and other signs of aging, skin organoids were irradiated for 20 min, twice, with a 2 h interval between exposures with a combination of UV-A and B, that closely mimic natural solar radiation. This procedure was repeated every two days, resulting in three UV exposures. The findings indicated skin barrier disruption, a notable increase in epidermal thickness and of the number of sunburn cells, identified by fragmentation or condensation of cell nuclei. Moreover, an increased skin pigmentation due to the higher number of melanocytes compared to control skin organoids, downregulation of genes related to skin barrier function (filaggrin and loricrin), and extracellular matrix degradation (significant decrease in type I collagen expression and an increase in MMP-1 expression) were observed. Of interest, a detrimental effect of UV radiation on hair follicles was described [70].

An important discovery highlights the potential of skin organoids in therapeutic applications, in particular for skin regeneration and wound healing, which are closely related to skin aging and are affected by the changes that occur in the skin over time [71]. Kim et al. generated skin organoids using iPSCs obtained from HLA-homozygous cord blood mononuclear cells and peripheral blood mononuclear cells with the aim of overcoming immune rejection, a significant hurdle in clinical applications of stem cell therapies. To test the functional abilities of the skin organoids, they were transplanted into immunodeficient mice with skin lesions. Following a good integration with the host tissue, they promoted healing, restoring normal skin functions [72].

Although organoids are defined as useful 3D skin models in vitro, there remain many issues to overcome. The main limitations include the absence of physiological communication between tissues, the incomplete development of vascularity, and impaired formation of complex neural networks and groups of immune cells that are often not fully integrated [73,74]. In addition, two specific limitations for organoids of aged skin are related to the low reprogramming efficiency in iPSCs of cells obtained from aged donors as compared to those of young subjects, and to the potential loss of some epigenetic markers of aging during the reprogramming process [75,76]. Furthermore, it is important to highlight that there is not currently a universally shared and standardized protocol for skin organoid generation, since organoids often exhibit high heterogeneity [77]. The possibility of having biobanks of organoids related to various pathologies could be a way to overcome this obstacle. However, the application of organoids in aging research holds great promise, enhancing our understanding of aging, identifying potential molecular markers for clinical evaluation of aging interventions, and suggesting strategies to combat aging and age-related diseases. Continued advancements in organoid technology and methods will provide a valuable tool for modeling the changes that occur with advancing age in human skin and for studying the development of age-associated skin conditions.

### 4.3. Reconstructed Human Skin (RHS)

The term “Reconstructed Human Skin” (RHS) refers to laboratory-grown skin models (dermal equivalent) mimicking the structure and function of human skin. These 3D models may consist of either just a skin layer or multi-layers (epidermis and dermis), depending on the cell type used and the proposed application. The RHS model is widely used in experimental research to study basic skin biology and disease mechanisms and to test pharmacological and cosmetic approaches. In light of this, we can identify the reconstructed human epidermis (RHE) model as an advanced in vitro 3D system that tries to replicate the fully differentiated epidermis without a dermal compartment. This model is composed by culturing only keratinocytes (from primary cultures or cell lines) on a supportive and inert matrix (i.e., collagen solution), allowing them to differentiate and create a multi-layered structure that is very similar to natural epidermis. Keratinocytes are plated into transwell chambers and shortly cultivated under submerged conditions; subsequently, the cultures are exposed to the air–liquid interface, which induces keratinocyte differentiation [78,79]. After two weeks of culture, a structure like the epidermis is characterized by classical cellular stratification (epithelium with basal, spiny, granular, and corneal layers), and the physiological properties of the human epidermis, that resembles a physiological epidermis. The RHE models are extensively used for dermatological research, including studies on barrier function, irritation, and drug permeability, and are used to test anti-aging treatments and products. They provide a more ethical and cost-effective alternative to animal testing and are valuable for understanding skin physiology and pathology. Several RHE are commercially available and supplied by various companies (listed in the review of Hofmann et al. [80]). Moreover, “pigmented RHE models”, which also incorporate melanocytes of different phototypes in the basal layer of the epidermal construct, can be either produced or purchased [25]. These systems had been utilized to study in a highly controlled environment the phototoxicity, pigmentation disorders, and test the effectiveness of commercially available cosmetic products such as sunscreens [81]. Hall and colleagues recently reported that pigmented RHE models can achieve realistic epidermal pigmentation through the process of melanocore transfer between melanocytes and keratinocytes in the epidermis [82]. The RHE model was also employed to investigate how the aging process can influence the amount, function, and microRNA (miRNA) content of the extracellular vesicles (EVs) released from the keratinocytes of young or aged skin samples. The results demonstrated that aged keratinocytes release more EVs compared to non-senescent keratinocytes; EVs from aged cells showed a specific microRNA profile characterized by an elevated level of miR-30a, central in the regulation of human epidermal barrier functions, and finally, inhibited the proliferation of young keratinocytes, and impaired the initial stages of wound healing in mice [83].

The RHS model has been improved by creating the full-thickness skin equivalent (FTSE). FTSE refers specifically to a type of RHS that replicates both skin layers (epidermal and dermal) simultaneously, thus providing a more complex model of human skin. FTSE is therefore formed by keratinocytes, fibroblasts, and extracellular components of the matrix, and it is for this reason that the FTSE model is used in wound healing studies, skin regeneration, and advanced dermatological research. Specifically, a keratinocytes’ layer is seeded on top of a fibroblast-loaded matrix and initially cultured under submerged conditions. After a short period, the culture is exposed to air, like the RHE protocol. Interestingly, skin aging can be induced in FTSE models by treatment with the cytostatic drug mitomycin C. Diekmann et al. developed and characterized a 3D skin model that accurately reflected the structural and functional aspects of aged skin. They created reconstructed skin co-culturing fibroblasts and keratinocytes on a collagen–glycosaminoglycan–chitosan scaffold mimicking the ECM, and aging was induced by the exposition of fibroblasts to mitomycin-C. This model exhibited key markers of aged skin, including increased expression of AGEs, decreased collagen I, and an increase in MMP-1 activity [26].

In summary, the literature documents the significant advancements and applications of RHS models in experimental research. The development of these sophisticated models allows for studying skin aging processes, thus providing a precious tool for understanding skin physiology and pathology and testing pharmacological and cosmetic treatments.

### 4.4. The Microfluidic Culture Device Called “Skin-on-Chip”

Nowadays, “Skin-on-Chip” (SoC) technology represents a significant advancement in the 3D skin models’ field. It overcomes the limitations of animal models by offering reproducible and reliable platforms for ex vivo modeling, ensuring the physiological relevance of in vivo skin [84]. In more detail, SoC is an advanced microfluidic culture device that integrates living cells into a micro-engineered environment to accurately mimic the dynamic environment, simulating the movement of fluids present in the human skin. Zhang et al. described this model as follows “Skin-on-Chip is to culture skin tissues within a microfluidic system, which can control many physical and biochemical parameters such as medium flow, mechanical force and gradients of biochemicals, mimicking the 3D microenvironments of the natural human skin” [85]. Key features of SoC devices are a microfluidic system that allows the continuous perfusion of fluids and a dynamic environment that mimics various physiological conditions such as mechanical stress, nutrient gradients, and fluid flow. Having the ability to adjust the physical parameters to control the cellular microenvironment, SoC technology has promoted the transition from static to dynamic 3D models that more accurately recapitulate human physiology, including the integration of vasculature and the immune system [84]. Constant and in-depth experimental research has enabled the creation of perfusable vascular channels in 3D skin-equivalent models (composed of normal human dermal fibroblasts and normal human epidermal keratinocytes). These models are developed by seeding endothelial cells into the dermal layer to induce vascularization, forming capillary networks [35].

Actually, few articles on the use of 3D aged skin models placed in microfluidic systems are available. Lim et al. induced aging in fibroblast/keratinocyte skin equivalents constructed on a microfluidic cell culture device using magnetic stretching with the aim of inducing aging in the skin equivalent and forming “Wrinkled Skin on the Chip” (WSOC) [27]. Briefly, a co-culture of human fibroblasts and keratinocytes was generated: a collagen solution containing fibroblast cells was placed in the SoC chamber in a CO_2_ incubator for 1 h to form a gel. Subsequently, to develop the stratum corneum of the epidermis, human keratinocytes were sprinkled onto the collagen layer and then incubated for 1 h for cell adhesion. Finally, the 3D model was subjected to uniaxial stretching using attractive and repulsive forces between an electromagnet and a permanent magnet. The mechanical stimulus reduced the proliferation of fibroblasts and keratinocytes and decreased the production of collagen, fibronectin, and keratin. Due to the lower production of these proteins, the skin equivalents could not maintain the stratum corneum or withstand the tensile stress of magnetic stretching, resulting in wrinkle formation [27]. WSOC can be effectively used to test the effect of anti-wrinkle formulations and cosmetics before or after the in vivo approaches.

In the work of Jeong et al., for the first time, an in vitro aged FTSE has been developed into an advanced SoC technology that used mechanical stimulus reflective of the circadian rhythm. Primary human fibroblasts were suspended for five days in collagen I solution to create the dermal layer; next, primary keratinocyte cultures were left to attach to the newly formed dermal layer before the air exposure phase, in which cells are induced to differentiate. To simulate the aging process, a periodic mechanical stimulation that mimicked the circadian rhythm for over 28 days was applied. This methodology induced aging in in vitro skin models, as observed by a reduced contraction, decreased epidermal layer thickness, and increased β-galactosidase expression. By combining an FTSE with dynamic mechanical stimuli reflecting the circadian rhythm, this study provided a more accurate and physiologically relevant platform for investigating skin aging [28]. Another study on a 3D model revealed the morphological changes in blood vessels induced by aging fibroblasts. The 3D model integrated human blood vessels, formed within the collagen gel matrix by human umbilical vein endothelial cells (HUVECs), into a pre-formed channel combined with young or senescent fibroblasts. Senescent fibroblasts exerted excessive traction stress on the surrounding ECM, leading to significant mechanical rearrangement of ECM fibers and promoting angiogenesis through the SASP phenotype. To confirm this result, rapamycin, an SASP inhibitor, counteracted the secretory phenotype of senescent fibroblasts, modulating their impact on blood vessels [29].

With promising applications in simulating the skin aging process and evaluating anti-aging treatments, these cutting-edge models offer new opportunities for more relevant studies of skin physiology and its pathological changes, owing to their ability to dynamically simulate human skin’s physiological and mechanical conditions. The main advantages and disadvantages/limitations of the SoC systems are listed in Table 1.

### 4.5. 3D Bioprinting Models for Skin Aging as a Viable Alternative to Traditional Animal Testing

A bioprinted model refers to a 3D structure created using bioprinting, an advanced technology that can form biological structures with a complex architecture closely resembling a physiological microenvironment. Bioinks, which are mandatory to build 3D models, are composed of living cells (such as fibroblasts, keratinocytes, adipocytes, endothelial cells, and melanocytes [90]), preferably derived from primary cultures and autologous stem cells [91] and growth factors [92] combined with biomaterials (aqueous formulations as a hydrogel). These biomaterials form a solid post-printing structure that mimics the extracellular matrix (ECM) and supports the cells within, ensuring their proliferation and differentiation. In recent years, significant progress has been made in developing and applying bioprinting technology and using organoids as a component of bioink. They recreate miniaturized tumor clusters with hierarchically organized cells, thus allowing a better simulation of the intrinsic characteristics of the tumor microenvironment [93]. Polymers used in bioinks can be natural or synthetic (such as collagen, dextran, fibrin, agarose–chitosan, gelatin, hyaluronic acid (HA), hydroxyapatite, alginate, methylcellulose, polylactic acid (PLA), polyglycolide (PGA), poly(lactic-co-glycolic acid) (PLGA), and polyethylene glycol (PEG) [94]). All these biological components are homogenously deposited on a substrate following the natural layer-by-layer distribution by different approaches, such as inkjet, extrusion, and laser-assisted technology [95].

Bioprinting technology has made significant strides in recent years, demonstrating the ability to create tissue constructs that closely replicate a range of tissues and organs, such as liver, kidney, bone, heart, skin, neurons, and vascular systems [95]. This advanced technology is also currently applied in cancer research [96]. For example, bioink loaded with lymphoid cells isolated from patients with chronic lymphocytic leukaemia has been recently established [97]. Moreover, in a very recent work, a new 3D bioprinting technique has been developed to recreate the natural structure of human corneal stroma using adipose tissue stem cells [98].

The 3D bioprinted skin, a human cell-based full-thickness skin model, has recently emerged as an advanced technology in tissue engineering, regenerative medicine, and the study of skin aging since it gives the potential to recreate complex tissue structures that closely resemble physiological skin with all dermal layers, blood vessels, nerves, muscles, and skin appendages (sebaceous, sweat, and mammary glands, hair follicles, nerves, and fingernails) [90,99,100,101]. It enables the precise placement of different cell types and biomaterials in a spatially organized manner, closely replicating the architecture of natural tissues. This innovative approach allows the creation of skin equivalents that always more accurately mimic the structural and functional characteristics of natural skin and that can be used as clinical-grade surgical grafts, for the development of topical formulations, dermal toxicology research, and for testing anti-aging therapies [102]. Lee et al. were the first to create a 3D collagen hydrogel structure that mimics stratified skin by bioprinting two distinct layers of cells: inner fibroblasts and outer keratinocytes [30].

Ansaf and colleagues have reported on the technical procedure to obtain a 3D bioprinting model [102]. A notable advantage of this approach was that the layer-by-layer deposition ensures obtaining a printed skin closely like the physiological organization of human skin. In fact, the first layer deposited is the epidermal one, composed of keratinocytes, and the dermal layer containing fibroblasts is the deepest layer. After printing, the newly formed skin construct is left to grow in a cellular-appropriate environment so as to promote cell–cell interaction to form functional tissue.

A very recent work generated an advanced immunocompetent 3D bioprinted human skin model to assess skin sensitization. The authors incorporated macrophages into photopolymerizable bioink created by blending silk fibroin methacrylate, gelatin methacrylate, and photoactivated human platelet releasate. The bioink was deposited within a transwell system to create the 3D skin construct, where an artificial basement membrane supported both an epidermal layer and a vascularized dermal layer. Cultured in an air–liquid interface, the printed construct showed a differentiated keratinocyte layer and dermal ECM remodelling by fibroblasts and endothelial cells, with the incorporation of macrophages further enhancing the model’s physiological relevance [103].

Even if 3D bioprinted skin is a very recent technology, the scientific literature reports on this approach employed for skin aging studies are growing [92,101,104,105]. Further studies are needed to develop 3D printed models specifically for studying skin aging. However, a recent review explored the role of 3D bioprinting to understand the mechanism of therapy-induced senescence (TIS) (or tumor dormancy) in cancer [106]. TIS is a particular condition in which both tumor and normal cells, in response to radiation and chemotherapy, become senescent. The accumulation of senescent cells in tumors can, paradoxically, promote tumor relapse, metastasis, and resistance to therapy due to the expression of the SASP phenotype [107]. The 3D bioprinting recreating a 3D structure that mimics the in vivo tumor microenvironment allows studying more deeply the cellular interactions between different cell types, including senescent and non-senescent tumor cells, within a realistic ECM.

A printed skin model made by a bioink, composed of keratinocytes, fibroblasts, and melanocytes, suspended in a biocompatible hydrogel matrix has been generated to obtain a 3D skin model showing a visible pigmentation. Melanocytes were sequentially printed onto the dermal layer along with keratinocytes to induce skin pigmentation and then the new skin equivalent was exposed to the air–liquid interface [31].

In summary, the advancements in 3D bioprinted skin models represent the most significant leap forward in tissue engineering and regenerative medicine, offering unprecedented capabilities to replicate natural skin’s complex structures and functions. Although the scientific literature is still emerging, these models show great promise in accurately mimicking physiological skin and studying its aging processes. The use of various bioinks, together with innovative techniques like integrating macrophages and assessing stimulus-induced senescence, highlights the potential of these models for both clinical applications and in-depth research. The continued development and refinement of these technologies will be crucial to deepen our understanding of skin aging and assessing therapeutic interventions.

**Figure 3:**
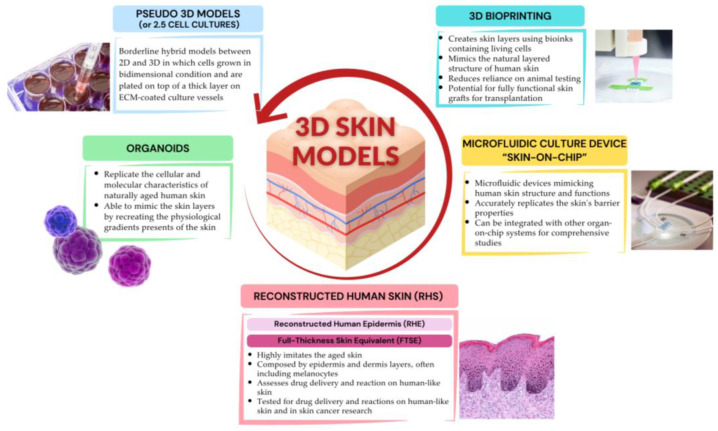
Highlights of 3D cellular models used in human skin aging research. Created with www.canva.com, accessed on 24 July 2024.

A summary of the highlights of 3D cell culture models used in human skin aging research is represented in Figure 3.

### 4.6. Future Directions for 3D Aged Skin Model Research: The Crucial Role of Microbiota in Enhancing the Realism and Functionality of 3D Skin Models

A very interesting advantage that should not be underestimated in the use of 3D skin models is the inclusion of the skin microbiota. The skin is colonized by several bacteria that can change significantly throughout life, forming a complex ecosystem with skin cells and closely interacting with the host’s immune system to regulate immune responses [108,109]. It is well known that the microbiota significantly impacts the biology of in vivo skin, contributing to the maintenance of the outermost layer of the epidermis, the stratum corneum, which results from specialized keratinocyte differentiation [110] and cornification processes. In this layer, the complex microbial ecosystem can promote keratinocyte differentiation and preserve the integrity of the healthy barrier [111]. The skin microbiota interacts with keratinocytes through different pattern-recognition receptor-dependent mechanisms (toll-like receptors (TLRs)), inducing the secretion of cytokines and other immune mediators [112]. Since the crucial role of the microbiota in the skin is absent in 3D models, it does not fully mimic skin physiology and skin–microbiota interactions. Establishing a realistic microbiota allows the understanding of the dynamic interactions between skin cells and microbes, even in long-term studies. Excluding the 2D in vitro models, which are inadequate for developing systems that can accurately replicate the relationship between all skin cells and microbiota since they lack stratum corneum and excluding the animal models characterized by their own microbiota not quite similar to those of human skin [113,114], recent advancements aim to establish and maintain microbiota onto more complex human skin models. Rikken and colleagues recently established a cost-effective methodology that is easily usable with 3D skin models with different cell types and bacteria, which is an alternative to experimental animal models in preclinical research [33]. Landemaine et al. treated a 3D model with *Staphylococcus epidermidis* as a single strain and with skin microbiota from a healthy individual and observed that colonization with a complete microbiota is less invasive, better tolerated, and promotes keratinocyte proliferation and cohesion more effectively than single strains, highlighting the importance of integrating the microbiota into 3D skin models to increase the soundness of the results [115]. Similar conclusions were later presented by Loomis et al., who used a commercial 3D skin tissue equivalent (EpiDerm) co-cultured at the air–tissue interface with bacteria isolated from swabs of healthy human skin. They found that the presence of a microbial community had a more significant effect (changes in epidermal thickness, epidermal cell proliferation, and filaggrin production) on human skin (epidermal layer) than individual taxa [116]. This result underlines the importance of a complete microbiota in studies involving the use of 3D models.

The benefits of in vitro 3D models with integrated microbiota could be significant in the field of dermatology, basic biology, and are valuable for testing the effects of drugs and cosmetic products. Moreover, 3D skin models using aged patient-derived cells and microbiota could represent a cutting-edge technique for developing and studying targeted, personalized interventions.

To the best of our knowledge, there is only one work investigating the influence of the microbiota in a 3D model of aged skin. Briefly, a commercially available human 3D skin full-thickness model composed of keratinocytes and fibroblasts was exposed to Polyinosinic-polycytidylic acid (Poly(I:C)), an agonist of toll-like receptor 3 (TLR3) and retinoic acid-inducible gene I (RIG-I)-like receptors), to induce aging of the skin model. Then, the aged 3D skin model was treated with supernatant derived from the *Streptococcus* cultures (*Streptococcus pneumoniae*, *Streptococcus infantis*, and *Streptococcus thermophilus*) collected from young women’s faces by sterilized skin tape. In the aged 3D model, the exposure to *Streptococcus* supernatants restored the skin barrier in terms of elasticity increase, hydration, a decrease in desquamation, collagen upregulation, and improved lipid synthesis. All these features could be attributed to spermidine, a polyamine secreted by *Streptococcus* cultures [32].

The skin microbial composition predicts chronological age in adults, as it is significant in aged subjects. A very recent article investigated the complex relationship between skin microbiota and the signs of skin aging. Using a comprehensive multi-study analysis, the authors identified specific microbial characteristics that correlate with various markers of aging on the skin [117]. However, they do not use 3D models. Future research incorporating patient-derived aged fibroblasts, immune cells, and host microbiota could create more sophisticated 3D skin models that reflect the individual characteristics of older patients, mimicking the physiological interactions between the epidermis, dermis, and immune cells.

In Table 2, the experimental works using different types of 3D skin models exclusively for the study of aging research are listed.

## 5. Conclusions

Three-dimensional in vitro skin models represent a significant advancement in the study of skin aging, providing a more accurate and comprehensive understanding of this complex process. Traditional 2D models, still widely used today, fail to replicate human skin’s intricate architecture and cell–cell interactions. Of note, 3D models, such as organoids, skin-on-chip, reconstructed human skin, and 3D bioprinting, offer experimental conditions closer to in vivo environments (Figure 2). Specifically, 3D models allow for more in-depth studies of physiological changes in the structure and function of the skin over time, including collagen degradation, reduced cellular vitality, and altered immune responses. Additionally, 3D models derived from aged patient cells could help develop new personalized anti-aging treatments. In addition, integrating skin microbiota into these models further enhances their realism and clinical translatability.

Despite promising advancements in this field, challenges remain, such as standardizing protocols, incorporating all relevant cell types, and recreating the exact physiological conditions of aged skin. Research and development are essential to overcome these obstacles and fully realize the potential of 3D skin models for studying aged skin. In summary, using advanced 3D skin models holds great promise for improving our understanding of skin aging and developing effective treatments, advancing scientific knowledge and clinical applications in the field.

## Figures and Tables

**Table 1:** Advantages and disadvantages and/or limitations of Skin-on-Chip technology.

SoC Advantages	Ref.	SoC Disadvantages and/or Limitations	Ref.
Reproducible and reliable skin systems	[84]	Highly complex to design and develop	[12]
Ensures a physiologically cell density by supplying oxygen and nutrients while removing waste through culture media perfusion	[86]	Excludes immune cells, hair follicles, and sweat glands	[87]
Better simulation of barrier function, epidermal thickness, keratinocyte differentiation, and immune response in in vitro skin models	[88]	Requires significant time and work investments	[12]
Substance transport is more physiologically relevant, allowing for a more accurate evaluation of parameters (i.e., molecule toxicity and delivery)	[89]	Mainly focuses on the skin excluding between organs	[84]

**Table 2:** Summary of experimental studies using 3D skin models in aging research.

Model	Structure and Cell Type	Culture Conditions	Stimuli to Induce Aging and Time to Establish Aged Model	End-Points	Ref.
**PSEUDO-3D** **SYSTEMS**	2.5D collagen model Human dermal fibroblasts (FF-95)	Collagen solution mixed with DMEM (10% FBS).	Replicative senescence. The entire process of cultivating fibroblasts, inducing senescence, and conducting experiments spanned several weeks. The exact duration is not explicitly detailed.	2.5-dimensional migration assay used to determine and compare speeds of migration and contraction of young and senescent dermal fibroblasts.	[49]
**ORGANOIDS**	3D organoid model composed of iPSC-derived keratinocytes. iPSCs	To differentiate iPSCs into keratinocytes: DKSFM medium supplemented with retinoic acid and BMP. At 4th day, medium was replaced with DKSFM in presence of EGF. iPSC-derived keratinocyte culture: DKSFM supplemented with EGF and Y27632 on the dish precoated with type I collagen and fibronectin. To generate organoid: fibroblasts were embedded in type I collagen gel in the 0.4 μm pores insert and cultured in DMEM for 1 week; keratinocytes differentiated from iPSCs were seeded on collagen gel and cultured for 2 days in DKSFM supplemented with EGF and ROCK inhibitor Y27632.	Organoids were irradiated with γ-rays using cobalt 60 as the radiation source. The dose rate was 0.44–2.61 Gy/min. Induction of iPSCs to keratinocytes from 14 up to 34 days. Construction of organoid on collagen hydrogel 14 days. Culturing the 3D skin model 1-2 weeks.	In keratinocytes present on organoids, the IR exposure enhanced senescence markers (p16 and p21) and g-H2AX foci formation. Application: skin function and DNA damage response after ionizing radiation exposure in 3D skin organoid.	[65]
	Model of sUV-exposed skin using human iPSC-derived skin organoids containing hair follicles. iPSCs	To establish skin organoid: on day 0, iPSCs seeded in essential medium with ROCK inhibitor, Y-27632, to generate EBs. At EB size of 250 μm, transfer of EBs into individual new ultra-low attachment plates in differentiation medium containing matrigel, and specific factors to initiate non-neural ectoderm induction. To induce cranial neural crest cell formation after 4 days, specific factors to prevent off-target chondrogenic differentiation were added. On day 12, to induce self-assembly of the epidermis: transfer all organoids into ultra-low attachment plates in skin organoid maturation medium.	sUV irradiation: 20 min, twice with a 2 h interval between exposures, resulting in a total of 50 kJ/m^2^ of sUV. These exposures were conducted at 2-day intervals for a total of three exposures. Generation of skin organoids 10–12 weeks. Induction of sUV damage 20 min, twice with a 2 h interval between exposures, for a total of three exposures conducted at 2-day intervals.	This model recapitulated several symptoms of photodamage, including skin barrier disruption, extracellular matrix degradation, and inflammatory response. Moreover, sUV induced structural damage and catagenic transition in hair follicles.	[70]
**RECONSTRUCTED HUMAN SKIN**	Reconstructed human epidermis (RHE). Normal young (<5 years) and aged (>60 years) human primary keratinocytes obtained from child foreskin or abdominal biopsies.	Keratinocytes grown submerged in the culture medium (DMEM-F12) for 72 h, then kept emerged at the air–liquid interface for 14 days in the medium (DMEM-F12 + CaCl2 supplemented with vitamin C) changed daily and supplemented with extracellular vesicles every 2 days.	Treatment with extracellular vesicles obtained from old keratinocytes’ culture medium. The entire process from cell seeding to the completion of the epidermal reconstruction took approximately 16 days.	Decrease in the tissue thickness. The changes in the intercellular communication mediated by extracellular vesicles occurring during aging process in keratinocytes could be involved in the functional defects observed in aged skin.	[83]
	3D skin equivalent. Fibroblasts and keratinocytes.	Co-culture of skin fibroblasts and keratinocytes on a collagen–glycosaminoglycan–chitosan scaffold.	Mitomycin C. The overall process, including seeding and culture, took approximately 14 days to achieve a fully differentiated 3D skin model.	Appearance of typical histological and biomolecular features of aged skin. This model, with comparable characteristics of in vivo aged skin, is proposed as a valuable tool to study the aging process and the effects of potential anti-aging formulations in a more physiological environment.	[26]
	Full-thickness human skin equivalent generated with early- and late-passage fibroblasts. Primary fibroblasts and keratinocytes isolated from surplus skin from cosmetic surgery.	Fibroblasts were cultured within a collagen matrix, overlaid with human keratinocytes to form a stratified epidermis, and maintained in standard culture conditions.	Replicative senescence of fibroblasts. Overall time was approximately three weeks.	Skin equivalents with late-passage fibroblasts had a thinner dermis than early-passage fibroblasts. The study provides evidence of dermal–epidermis crosstalk and insight into how aging fibroblasts affect skin structure.	[118]
	Human skin equivalent. Primary human dermal fibroblasts and keratinocytes.	Young or senescent human dermal fibroblasts (*senoskin*) were seeded in a collagen gel. Keratinocytes were laid on top of fibroblasts and were then lifted to the air–liquid interface to start differentiation on day 3.	H_2_O_2_ and doxorubicin. After 10 days, the skin equivalents were harvested. The overall process, including seeding and culture, took approximately 2–3 weeks.	The use of senescent fibroblasts in the 3D skin model led to significant structural alterations (thinning of the epidermal layer and a reduction in dermal collagen) that mimic those observed in aged human skin.	[40]
	Full-thickness human skin equivalent. Primary human dermal keratinocytes isolated from skin biopsies of healthy adult donors.	Primary human dermal keratinocytes were embedded in collagen I matrix, keratinocytes were seeded on top and submerged for 7 days. The keratinocytes were placed at the air–liquid interface for 7 days. Then, the skin equivalents were treated with the extract of *Solidago alpestris.*	Replicative senescence and H_2_O_2_. The time needed to establish the 3D aged model was not specified in detail.	The extract of *Solidago alpestris* exhibited weak senolytic activity and reduced hallmarks of senescence. Treatment with the extract led to increased cell proliferation and the maintenance of epidermal thickness in the 3D skin models, suggesting a protective effect against age-related thinning of the skin.	[39]
	Full-thickness human skin equivalent. Primary fibroblast and keratinocytes.	Fibroblasts were cultured in a collagen matrix to form the dermal layer, overlaid with keratinocytes to form the epidermal layer. The culture was maintained at the air–liquid interface to promote stratification and differentiation of the epidermal layer.	Aging was induced by manipulating the expression of p16INK4A, a key regulator of cellular senescence. Although if time to obtain a 3D aged model is not clearly specified, it can be assumed that the overall process could take about 2–3 weeks.	Overexpression of p16INK4A in keratinocytes induced the aging phenotype, characterized by reduced cell proliferation and alterations in the skin structure, such as thinning of the epidermal layer. Silencing p16INK4A led to a loss of the aging phenotype.	[119]
	Full-thickness human skin equivalent. Human keratinocytes and human dermal fibroblasts isolated from abdominal skin derived from plastic surgery.	Cells were treated with Tert-butyl hydroperoxide (tBHP) twice a day for 4 days and cultured for 9 days. Fibroblasts were mixed with collagen matrix. Keratinocytes were placed over the dermal layer and grown in submersed conditions for 48 h. Skin equivalents were cultured to air–liquid interface in differentiation media with ascorbic acid, transferrin, and CaCl2 for 7 days.	tBHP, an inducer of oxidative stress and cellular damage. The overall process, after initial cell seeding, took approximately 2 weeks.	tBHP could serve as a valuable agent for modeling the effects of environmental pollutants on skin aging and for assessing potential anti-aging therapies.	[120]
	Commercial full-thickness skin model. Human keratinocytes and human dermal fibroblasts.	The skin was treated with Resveratrol nanoliposomes (Res-NLPs).	Hydrogen peroxide (H_2_O_2_) or UV irradiation. The detailed timeline beyond the general 2–3 weeks for the 3D skin model was not mentioned.	Resveratrol nanoliposomes significantly enhanced skin care benefits by improving antioxidant capacity and promoting collagen synthesis.	[121]
	Skin equivalents. Human foreskin fibroblasts (HFFs).	Fibroblasts, after transfection with siRNA, were suspended in the collagen solution and allowed to polymerize at 37 °C.	Growth differentiation factor 15 (GDF15) knockdown in HFF induced premature senescence associated with mitochondrial dysfunction. The overall process, after initial cell seeding, took approximately 1–2 weeks.	GDF15 knockdown triggered mitochondrial stress, inducing senescence and reducing epidermal thickness in skin equivalents. This model allows mimicking the in vivo conditions of skin aging and assessing the role of GDF15 in maintaining cellular and mitochondrial health.	[122]
	Commercial full-thickness skin model, which included both dermal and epidermal components.	Skin model was topically treated with a standardized extract of *Kaempferia parviflora* (BG100) daily for six days.	UV exposure. Because a commercial skin model was used, the time needed to create the aged model is that of UV exposure (exposure daily for five days).	BG100 could be a promising component for anti-aging formulations, as it supports skin structure and reduces oxidative stress induced by UV exposure.	[123]
	Commercial full-thickness skin model, which included dermal fibroblasts and keratinocytes.	The skin was treated with *rosa gallica* extract for 1 h before UVB irradiation and then irradiated with UVB twice a day for 8 days.	UVB radiation. The overall process took approximately 10 days.	*Rosa gallica* extract reduced wrinkle formation, prevented collagen degradation, and downregulated COX-2 and MMP-1 expression in the skin model by targeting the c-Raf/MEK/ERK signaling pathway, effectively blocking UVB-induced aging effects.	[124]
	Human skin equivalent (HSE). Human dermal fibroblasts (HFFs) were collected from newborn foreskin and human keratinocytes.	HFF were mixed with type I collagen for 7 days, keratinocytes were allowed to grow inside low calcium epidermal growth media for 2 days and then inside normal calcium media for an additional 2 days. The HSE was cultured to the air–liquid interface for 7 days before use. HSE was treated with lutein and γ-tocopherol, key antioxidants found in pistachios.	UVA radiation. The overall process took approximately 18–20 days.	Pistachio antioxidants maintained the thickness and organization of the skin model after UVA exposure and preserved fibroblast morphology, offering a protective effect against morphological changes caused by UVA radiation.	[125]
	Commercial human 3D skin culture system. Human dermal fibroblasts and keratinocytes.	The skin was incubated with unripe peach (YPE) extract and UVB irradiated.	UVB radiation. Because a commercial skin model was used, the time needed to create the aged model is that of UVB exposure (at day 2, 4, and 7).	YPE could exert a protective role against UVB-induced skin aging focusing on maintaining the structural integrity of the skin by preserving key components like collagen XVIII.	[126]
**SKIN-ON-CHIP**	Wrinkled skin-on-chip (WSOC) developed by cyclic uniaxial stretching. Human fibroblasts and human keratinocytes cultured in DMEM with 10% FBS and KGM, respectively.	To form the structure of the WSOC, two PDMS layers were fabricated and combined. The collagen layer was formed in the cell chamber by adding a collagen solution containing fibroblasts. To form the stratum corneum of the epidermis, keratinocytes were sprayed onto the collagen layer of the WSOC. The WSOC was incubated for 1 h in a CO_2_ incubator for cell attachment. Every day for 4 days, to keratinocytes on the chip were given fresh KGM and the fibroblasts on the chip were perfused with fresh DMEM through the microchannels of the WSOC. The cells on the WSOC were exposed to air for differentiation.	Attractive and repulsive forces between a permanent magnet embedded in the wall of the cell culture chamber and an electromagnet outside the wall uniaxially stretched the skin equivalent at 5.3 mm/s. WSOC fabrication time 2 h. Fibroblasts were cultured for 4 days within the WSOC. Keratinocytes were then sprayed onto the collagen layer containing fibroblasts and incubated for another 4 days. The WSOC was subjected to uniaxial stretching for 12 h per day for 7 days. The entire process took approximately 2 weeks.	The stretching decreased the proliferation of fibroblasts and keratinocytes, resulting in lower collagen, fibronectin, and keratin production. Owing to the lower production of these proteins, the skin equivalents were not able to maintain their stratum corneum and withstand the tensile stress applied via magnetic stretching, resulting in the formation of wrinkles. Application: WSOCs can be used to test the effect of anti-wrinkle ingredients and cosmetics prior to in vivo experiments.	[27]
	Flexible skin-on-a-chip (FSOC). The FSOC comprises an upper and lower PDMS chips with a porous member within. In the centre of the FSOC, there is a culture chamber to cultivate cells. Primary human fibroblasts and primary human keratinocytes.	To obtain the dermal layer, the fibroblasts were mixed with type I collagen solution (5 days). To obtain the epidermal layer, the keratinocytes were attached to the dermal layer (2 days) and exposed to the air–liquid interface (3–28-days).	Mechanical compression stimulation (that reflects circadian rhythms) was applied to a 3D skin equivalent to produce an aging skin model. The entire process took approximately 35–37 days (including the initial setup and the 28-day stimulation period).	Aged full-thickness skin equivalent model that uses mechanical stimulus reflective of the circadian rhythm. Application: this model could be useful for conducting in vitro drug efficacy assessments and investigating new cosmetics.	[28]
	3D microfluidic polydimethylsiloxane (PDMS)-based chip consisted of microvessel surrounded by a collagen gel with embedded young or senescent skin fibroblasts. Primary human skin fibroblasts (HCA2) and primary human umbilical vein endothelial cells (HUVEC).	Senescent fibroblasts were embedded in a collagen solution and placed in the PDMS-based chips that were incubated at 37 °C for 1 h to enable gelation of collagen. HUVEC suspension was inserted into the channel and left to attach onto the collagen scaffold at 37 °C for 15 min.	Irradiation with 10 Gy of γ-ray. Although if time to obtain a 3D aged model is not clearly specified, it can be assumed that the overall process could take about 2 weeks.	Senescent, but not young, fibroblasts had the ability to induce sprouting angiogenesis. Senescent fibroblasts induced a pro-inflammatory environment due to their secretion of the senescence-associated secretory phenotype (SASP). This inflammatory milieu contributed to the dysfunction of endothelial cells. Senescent fibroblasts were able to mechanically rearrange the ECM fibers.	[29]
	Customized microfluidic device based on cyclic olefin polymers (COPs) that allows inserting the embedded fibroblasts into a chamber through a porous membrane to a second chamber where keratinocytes were grown. Human dermal fibroblasts and human immortalized keratinocytes (HaCaT).	Human dermal tissues from aged human cadavers were decellularized to remove all cellular components while preserving the ECM structure. The decellularized ECM was lyophilized and then reconstituted in a solution to form a hydrogel (as a scaffold for 3D skin culture). Fibroblasts were embedded in aged decellularized dermal extracellular matrix (dECM) hydrogels and HaCaT cells were seeded on top of hydrogels for 4 days and then cultured in an air–liquid interface (12 days).	dECM from aged human cadavers (70–90 years of age). The entire process took approximately 3–4 weeks.	The human dECM hydrogel, preserving essential components of the native human dermis, could be an efficient scaffold for dermal fibroblasts in a skin aging-on-a-chip model. The model offers a realistic environment for studying skin aging.	[127]
**3D BIOPRINTING MODELS**	3D bioprinted skin model used to study the impact of skin microrelief on the mechanical properties and aging process of the skin. No cells used. Mixed solution of gelatin and methacrylic anhydride to form Gelatin Methacryloyl (GelMA). Printing ink formed by addition to GelMA of lithiumphenyl-2, 4, 6-trimethylbenzoyl phosphinate (LAP) initiator and the light absorber.	Protocol used for generating the 3D model: - Digital Light Processing technology, a printing method enabled precise fabrication of skin microrelief patterns, crucial for studying skin aging. - Voronoi algorithm allowed the generation of accurate mesh formations, replicating the natural polygonal patterns found in human skin to create the microrelief models of different age groups. With these approaches, three groups of skin samples (young, middle-aged, and aged) were generated.	The aged skin model was constructed using the 3D modeling software from human skin images of aged subjects. The entire process, from GelMA preparation to the creation and printing of the skin microrelief models, spans over one week but the specific time was not detailed.	Skin samples with different microrelief levels were found to have different mechanical properties, highlighting the importance of these surface structures in maintaining skin elasticity. Three-dimensional bioprinting technology to create models that mimic the surface topography of skin at various ages.	[105]
	Full-thickness skin wrinkle model created using a 3D printer with acrylonitrile–butadiene–styrene (ABS) as the printing material. Human neonatal dermal fibroblasts and human neonatal epidermal keratinocytes.	The dermal mixture was prepared by mixing type I collagen, reconstruction buffer, fibrinogen, aprotinin, and fibroblasts. Then, thrombin was added to initiate the fibrinogen polymerization. To construct the epidermis, keratinocytes were seeded on the dermis layer and cultured for 10 days in the air–liquid interface.	Wrinkles created in the dermal layer during the collagen gelation process mimic aging skin. Retinoic acid, an anti-aging compound, was used to evaluate changes in skin wrinkles. The time required to print the model was not detailed.	The depth and width of the wrinkles in the skin models were measured using Swept Source-Optical Coherence Tomography (SS-OCT). This technology allows for detailed imaging of the skin model’s surface and subsurface layers, providing precise measurements of the effects of anti-aging treatments.	[128]
**3D AGED SKIN MODEL** **WITH MICROBIOTA**	Normal human 3D skin model at full-thickness (Epiderm-FT). Normal human keratinocytes and normal human dermal fibroblasts.	The tissue was cultured in DMEM containing gentamicin B, amphotericin B, and growth factors.	To induce aging, 3D skin models were treated with poly I:C, an agonist of TLR3 and RIG-I-like receptors. The aged 3D skin model was treated with supernatant derived from the Streptococcus cultures (*Streptococcus pneumoniae, Streptococcus infantis*, and *Streptococcus thermophilus*) collected from young women’s faces by sterilized skin tape. The overall process took place over 28 days.	This study shows the ability of Streptococcus supernatants to restore the skin barrier in terms of elasticity, increase hydration, decrease desquamation, upregulation of collagen, and improve lipid synthesis. All these features could be attributed to spermidine, a polyamine secreted by Streptococcus cultures.	[32]

FBS = Fetal Bovine Serum; iPSC = induced pluripotent stem cells; DKSFM = defined keratinocyte serum-free medium; BMP = bone morphogenetic protein; DMEM = Dulbecco′s Modified Eagle′s Medium; EGF = epidermal growth factor; IR= ionizing radiation; EBs = embryoid bodies; sUV = solar ultraviolet radiation; KGM = keratinocyte growth medium; PDMS = polydimethyl siloxane; poly I:C = polyinosinic-polycytidylic acid; RIG-I= retinoic acid-inducible gene I; TLR3= toll-like receptor 3.
